# Determining the optimal number and location of cutoff points with application to data of cervical cancer

**DOI:** 10.1371/journal.pone.0176231

**Published:** 2017-04-27

**Authors:** Chung Chang, Meng-Ke Hsieh, Wen-Yi Chang, An Jen Chiang, Jiabin Chen

**Affiliations:** 1 Department of Applied Mathematics, National Sun Yat-sen University, Kaohsiung, Taiwan, ROC; 2 Department of Obstetrics and Gynecology, Kaohsiung Veterans General Hospital, Kaohsiung, Taiwan, ROC; 3 Institute of Biomedical Science, National Sun Yat-sen University, Kaohsiung, Taiwan, ROC; 4 Multidisciplinary Science Research Center, National Sun Yat-sen University, Kaohsiung, Taiwan, ROC; 5 Da-Yeh University, Changhua, Taiwan, ROC; Taipei Medical University, TAIWAN

## Abstract

It is often helpful to classify biomarker values into groups of different risk levels to facilitate evaluation of a biological, physiological, or pathological state. Stratification of patients into two risk groups is commonly seen, but there is always need for more than two groups for fine assessment. So far, there are no standard methods or tools to help decide how many cutoff points are optimal. In this study, we developed a comprehensive package that included methods to determine both the optimal number and locations of cutoff points for both survival data and dichotomized outcome. We illustrated workflow of this package with data from 797 patients with cervical cancer. By analyzing several risk factors of cervical cancer such as tumor size, body mass index (BMI), number of lymph nodes involved and depth of stromal invasion, in relation to survival and clinical outcome such as lymph nodal metastasis and lymphovascular invasion, we demonstrated that the best choice for BMI and stromal invasion was two cutoff points and one for the others. This study provided a useful tool to facilitate medical decisions and the analyses on cervical cancer may also be of interest to gynecologists. The package can be freely downloaded.

## Introduction

Biomarkers have increasingly wide application in disease diagnosis, monitor of disease progression, assessment of prognosis, and development of pharmaceutical agents. Biomarkers are usually represented by continuous values and ordinal numbers, but in practice, it is often helpful to classify biomarker values into groups of different risk levels to facilitate evaluation of a biological, physiological, or pathological state. A convenient tool to find an optimal cutoff point is, therefore, of high interest. Multiple methods are available to determine a cutoff point, from the mean or median value of diagnostic biomarkers to methods based on distribution of values or association with clinical outcomes, such as the minimal p-value and maximization of combined sensitivity and specificity [1,2]. There has also been effort on developing a comprehensive tool to facilitate a systematic approach for cutoff determination [3].

While many studies have been devoted to find one optimal cutoff, there is often need in practical medicine to make not one, but two or even more cutoffs to stratify patients into several groups for fine assessment of risk and treatment plans. For example, a very low dose of a drug may not be effective at all, while an excessively high dose of the same drug may pose a disastrous side effect, and therefore, two cutoff points are needed to find the optimal dose interval for the drug. Tentative efforts have been made to construct statistical tools for multiple cutoff points, for example, X-Tile is a free tool that analyzes survival data for optimizing multiple cutoffs using a minimal p value approach, and an R package was also developed for dichotomized outcome [4,5].

Although the previous efforts were targeted on multiple cutoff points, they all assumed that the number of cutoffs had already been known or decided, and a straight-forward way to facilitate choice of an optimal number of cutoffs still remains elusive. In addition, a comprehensive tool that addresses both survival and dichotomized outcomes are also of interest to researchers. This study was, therefore, aimed to construct a statistical package to help decide the optimal number and location of cutoff points of any given biomarker in relation to survival data as well as dichotomized clinical outcomes, and to deliver it in an easy-to-use package that can be freely accessible to researchers who are interested. At the same time, examples were provided in this study to illustrate the workflow of implementing the package. By using information from cervical cancer for stratification of patients, we also aimed to address clinical concerns in cervical cancer by analysis of several clinicopathologic factors.

## Methods

**Fig 1:**
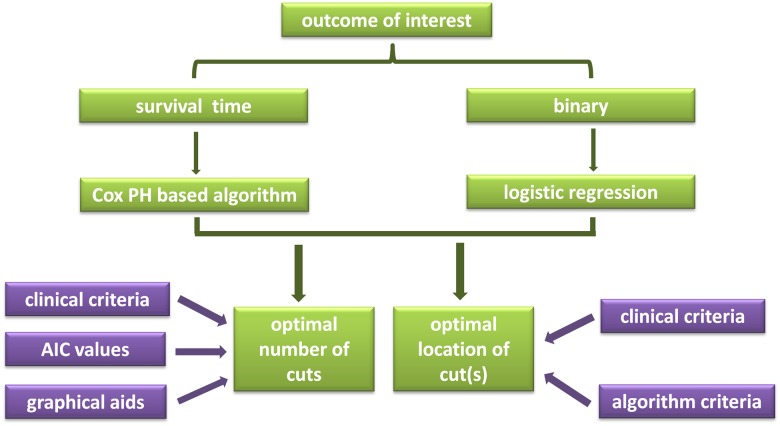
Work flow of deciding the optimal number and locations of cutoff points.

All the analyses in this study were performed with the statistical computing and graphic drawing language, R [6]. The workflow of implementing the package provided here can be found in Fig 1. Two R packages, histSpike and rms, were used for histogram and monogram drawing, respectively.

### The AIC method to determine optimal number of cutoff points

As the number of cutoff points, or equivalently, the number of risk groups, increases, the flexibility of the model also increases. At the same time, the number of parameters that needs to be estimated also increases. Akaike information criterion (AIC) can take into account both the model’s flexibility and complexity. By minimizing the AIC values, the optimal number of cutoff points can be determined.

We provided an R function (findcutnum) to calculate AIC values for both survival and binary outcomes, the codes of which can be accessed at http://www.math.nsysu.edu.tw/~cchang/cutoff/findcutnum.txt.

### Graphical aid of choosing optimal number of cutoff points

An R package, smoothHR, was used to plot a variable in question against outcome of interest to visually assist with the choice of the optimal number of cutoffs [7].

### Finding the location of cutoff point(s)

Assume there are K + 1 risk groups (i.e. K splits or cutoff points) for a continuous risk factor Z, then based on its values Z can turn into an ordinal variable Z* with K + 1 levels that can be represented by a group of K binary (dummy) variables Z_1_,…, Z_K_.

#### Survival data

Given the ordinal covariate Z*, the hazard function for the Cox proportional hazard (PH) model can be expressed as follows:

$$\begin{array}{l} h\left(t|Z^{*}\right)=h_{0}\left(t\right)e^{\sum_{k=1}^{K}\beta_{k}z_{k}} \end{array}$$ (1)

where h(t| Z*) is the hazard at time t given the risk factor Z, β_1_,…, β_K_ are the regression coefficients, and h_0_(t) is the baseline hazard at t. Cox PH regression model is executed using the function Coxph from the R package [8]. The optimal cutoff points are defined as the ones with the most significant (likelihood ratio test or log-rank test) split.

#### Dichotomized outcome

Given the covariate Z*, the log odds for the logistic regression model can be expressed as follows:

$$\text{ln}\left(\frac{p\left(Y=1|Z^{*}\right)}{1-p\left(Y=1|Z^{*}\right)}\right)=\overset{K}{\underset{k=1}{\sum^{\;}}}\beta_{k}Z_{k}$$ (2)

where p(Y = 1|Z*) is the probability that the response variable is equal to 1 given the risk factor Z* and β_1_,…, β_K_ are the regression coefficients. Logistic regression is executed using the function glm from R. The optimal cutoff points are determined by two criteria.

Criterion 1: significance. The optimal cutoff points are defined as the ones with the most significant (likelihood ratio test) split, similar to the survival data.

Criterion 2: maximum area under curve (AUC). Each set of cutoff points corresponds to a unique operating characteristic curve (ROC), and the optimal cutoff points are chosen to be the ones that maximize the corresponding area under the ROC curve.

Note that AUC is often used as a measure of classification quality of models. Therefore, maximizing AUC is similar to maximizing the classification quality of models.

All codes and instructions can be found at https://osf.io/ef7na/#.

### Patients and clinicopathological factors

Information of patients with cervical cancer, treated in KSVGH from January, 1990 to the end of 2012, was collected in the Kaohsiung Veterans General Hospital (KSVGH), a public teaching hospital in south Taiwan. The study was approved of by the Kaohsiung Veterans General Hospital Institutional Review Board (KSVGH IRB, protocol number: VGHKS16-CT8-06) and conformed to the current ethical principles of the Declaration of Helsinki. Written consents were obtained from all the patients.

Several variables concerning patients’ baseline and clinical characteristics, i.e., age, stage and histological types, were included in multivariate analyses. Age was included as a continuous variable, stage as a binomial variable as early (IA through IIB) and late (above IIB), and histological types as squamous cell carcinoma, adenocarcinoma and others.

## Results

### Patients with cervical cancer

Cervical cancer represents a major public health problem worldwide, as the third most common female cancer ranking after breast and colorectal cancer [9]. In this study, information of 797 patients with cervical cancer was collected. The median age of these patients was 54.6 years, and the median progression-free survival (PFS) and the overall survival (OS) were 3.4 years and 3.6 years, respectively. Most of them were at an early stage: 60.6% at FIGO stage IA through IB and 21.8% at IIA through IIB. Less than 20% of the patients had disease relapse, and 11.42% of all the patients died during the study period. Basic characteristics of the patients are summarized in Table 1.

**Table 1:** Distribution of the patients’ baseline characteristics.

Characteristics	Median (range)
Age, years	54.6 (23–100)
BMI*	24.25 (12.67–48)
Tumor size, cm	3.5 (0–17)
PFS*, years	3.39 (0.1–22.0)
OS*, yeas	3.57 (0.1–22.0)
FIGO* Stage	N (%)
IA1	39 (4.89)
IA2	10 (1.25)
IB	20 (2.51)
IB1	281 (35.26)
IB2	134 (16.82)
IIA	9 (1.13)
IIA1	37 (4.64)
IIA2	32 (4.01)
IIB	96 (12.05)
IIIA	15 (1.88)
IIIB	45 (5.65)
IVA	22 (2.76)
IVB	27 (3.39)
unknown	30 (3.76)
Histological type	N (%)
squamous cell carcinoma	571 (71.64)
adenocarcinoma	108 (13.55)
adenosquamous carcinoma	16 (2.01)
small cell carcinoma	5 (0.63)
Others^#^	14 (1.76)
unknown	83 (10.41)
Relapse	119 (14.93)
Death	91 (11.42)

* BMI: body mass index; PFS: progression free survival; OS: overall survival; FIGO: International Federation of Gynecology and Obstetrics.

^#^ 4 undifferentiated, 2 clear cell carcinoma, 2 lymphoepithelioma, 1 endometrioid, 1 large cell neuroendocrine carcinoma, 1 leiomyosarcoma, 1 malignant melanoma, 1 sarcoma botryoides, and 1 stromal sarcoma

### Single optimal cutoff point in relation to survival data

In order to stratify patients into groups of low risk and high risk, an optimal cutoff point is needed. Several methods are available to find this point in the package provided here to serve different outcomes of interest. One kind of outcome is associated with survival time, i.e., the elapsed time from diagnosis to event such as death and disease relapse. In survival studies, the subjects who were lost during follow-up are also accounted for as censored data. The Cox PH algorithm is usually used to analyze parameters in their association with survival time.

Lymph node metastasis is an important prognostic factor in cervical cancer [10]. In this study, we analyzed the 797 patients with cervical cancer for the association of the number of metastatic lymph nodes and disease relapse as well as death. The patients were randomly divided into two groups, with 497 patients as the training cohort and 300, testing. As shown in Table 2, two methods, log rank test and likelihood ratio test, were used to find the optimal cutoff point in the training set, and both chose 5 in both events, suggesting that patients with 5 or more lymph nodes affected by cancerous cells were at a high risk for disease relapse and death, while those with fewer, low risk. Validation was subsequently performed with the testing cohort of 300 patients in a multivariate analysis with age, stage, number of nodes and histological types. The results showed that advance stage and more than 5 metastatic lymph nodes independently associated patients with high risk for disease relapse and the subtype of squamous cell carcinoma independently put patients with low risk for relapse, while in the event of death, only the number of metastatic lymph nodes was significant (Table 2).

**Table 2:** Optimal cutoff point: Total number of lymph node metastasis vs. survival.

Training (n = 497)			Testing (n = 300)					
Methods	Cutoff point			Recurrence				Death
	Recurrence	Death		NMLN*	Stage	Histology		NMLN*
Log-rank test	5	5	HR*	6.653	163.2	1	0.117	5.781
						2	0.283	
Likelihood ratio test	5	5	p	0.007	0.0004	1	0.004	0.035
						2	0.281	

* NMLN: number of metastatic lymph nodes, HR: hazard ratio. HR1 for histology was the hazard ratio of squamous cell carcinoma vs. other types, and HR2, adenocarcinoma vs. other types.

### Single optimal cutoff point in relation to dichotomized outcome

Another common form of outcome in medicine is represented by presence or absence of clinical manifestations, for example, whether or not a patient has lymph node metastasis. Such dichotomized outcome relies on logistic-regression based algorithms to find the optimal cutoff. In this study, the likelihood ratio test and the maximal AUC test were used.

In cervical cancer, the size of the tumor is one of the criteria for staging patients [11]. We analyzed the relation between tumor size and presence of lymph node metastasis, and as in the above example, the patients were also randomly divided into a training cohort of 497 patients and a testing cohort of 300. Both the likelihood ratio test and the maximal AUC test found that a tumor size at or larger than 3.25 centimeters associated the patient with high risk for lymph node metastasis, while a tumor size smaller than 3.25 centimeters, low risk (Table 3). To validate the result, we performed a multivariate analysis with the testing cohort of 300 patients, where tumor size was included as a binary variable with 3.25cm as the cutoff point side by side with several other variables, i.e., age, stage and histological types. In response to presence of lymph node metastasis, only tumor size was a significant factor.

**Table 3:** Optimal cutoff point of tumor size against risk of lymph node metastasis.

Training (n = 497)		Testing (n = 300)			
Methods	Cutoff (cm)	Odds ratio	p value	AUC	Accuracy
Likelihood ratio test	3.25	5.881	<0.0001	0.700	0.674
Maximum AUC	3.25				

### Multiple cutoff points in relation to survival data

In clinical practice, it is often of interest to stratify parameters into more than 2 groups for fine assessment. The Cox PH based methods can still be used in survival studies to find multiple cutoff points, and logistic regression based methods in binomial outcomes. Additionally, we also provided an algorithm to calculate the AIC values and a graphical tool to facilitate the choice of cutoff numbers in case there are no obvious clinical arguments for the optimal number.

**Fig 2:**
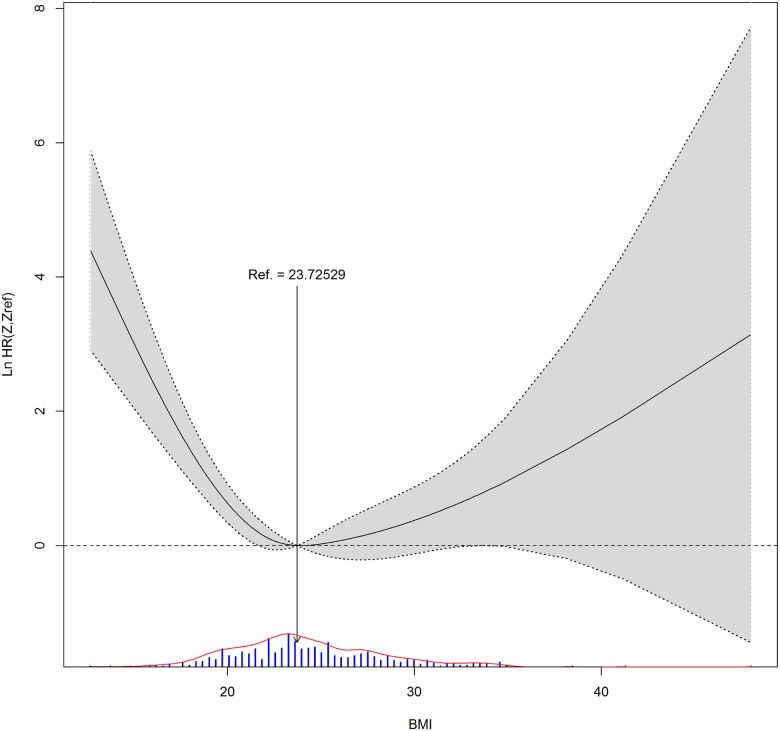
BMI distribution against hazard ratio (HR) in the event of death. Arrow marks position of the lowest HR. BMI histogram is illustrated with bars at the bottom of the figure.

Body mass index (BMI) is a good example of multiple cutoffs. As illustrated in the workflow in Fig 1, we took account clinical evidence, AIC values and graphic visualization to decide how many cutoffs were the best. BMI has been associated with many diseases, and a high BMI may put one at risk for poor prognosis [12,13]. At the same time, an extremely low BMI may not be very healthy, either [14]. Therefore, two cutoffs seem more appropriate than one. We then looked at the AIC values in case of BMI in relation to OS, which were 749.9 with one cutoff and 744.9 with two, also favoring two cutoffs to one. Moreover, when BMI distribution was plotted against hazard ratio (HR), shown in Fig 2, the lowest HR occurred when BMI was around 23, and any value bigger or lower than it showed a higher HR.

**Fig 3:**
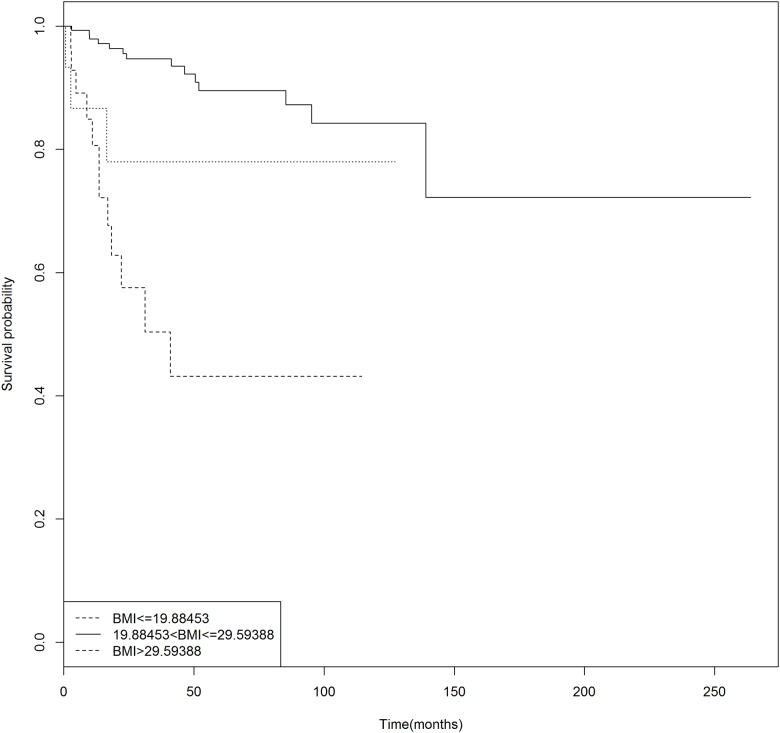
Kaplan-Meier curves for the three risk groups of BMI using the validation cohort of 300 patients. The log-rank test showed significant difference in risk of death between the groups (p = 0.028).

**Fig 4:**
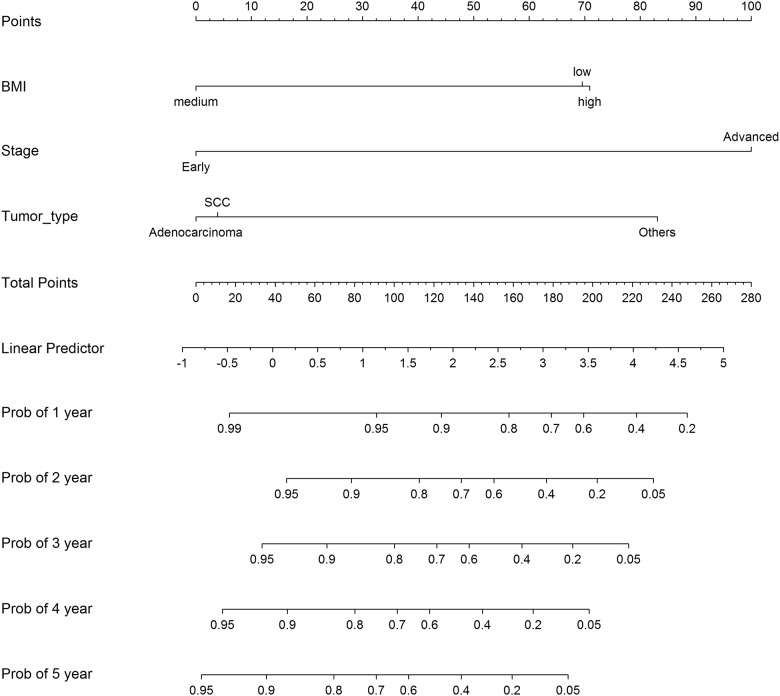
Nomogram of survival probabilities with independent risk factors using the validation cohort of 300 patients. Survival probabilities after 1 year through 5 years are calculated based on the effects of the three risk factors.

We thus decided that the optimal number of cutoffs for BMI was 2, and the log rank test and the likelihood ratio test were subsequently employed to locate the cutoffs. Again, the patients were randomly divided into a training cohort of 497 patients and a testing cohort of 300. Both the methods placed the two cutoffs at 19.88 and 29.59 (Table 4). The patients could be thus divided into three risk groups: those with BMI between 19.88 and 29.59 were at a low risk of death and those above 29.59 and below 19.88, high risk. The stratification of risk groups can be further visualized in Fig 3 where their Kaplan-Meier curves also show distinctive survival risks. We also validated the result with the testing set of 300 patients in multivariate analysis where other factors, age, stage, and histological types, were also included. The multivariate analysis showed that BMI, stage and histological types were independent risk factors of death (Table 4). The nomogram based on these factors is given in Fig 4, where survival probabilities after different numbers of years can be evaluated.

**Table 4:** Multiple cutoff points: Body mass index (BMI) vs. death.

Training (n = 497)			Testing (n = 300)									
Methods	Cut 1	Cut 2	BMI				stage		Histological types			
Log-rank test	19.88	29.59	HR1*	p 1	HR2*	p 2	HR*	p	HR1*	p 1	HR2*	p 2
Likelihood test	19.88	29.59	4.62	0.006	4.76	0.05	9.03	0.0002	0.175	0.002	0.161	0.043

*HR: hazard ratio. HR1 for BMI is the hazard ratio of low BMI over medium, and HR2, high over medium; HR1 for histological types is the hazard ratio of squamous cell carcinoma over other types, and HR2, adenocarcinoma over others.

### Multiple cutoff points in relation to dichotomized outcome

Multiple cutoffs can also be made if the outcome of interest is dichotomized. In cervical cancer, depth of stromal invasion measures how deep tumor invades the cervix stroma, and a fraction of 1/3 or higher puts patients at risk of poor prognosis [11]. In this study, we wondered what would be the risk if the cervical stroma was completely penetrated by tumor (fraction = 1). We, therefore, tested the invasion fraction for its optimal number of cutoffs as a covariate of presence of lymphovasular space invasion (LVSI), which was an important prognostic factor in cervical cancer.

First, the AIC value for one cut was 503.6, and for two, 491.1, favoring the latter. Second, as shown in Table 5, the odds ratio (OR), when two cutoffs were placed, of group 3 over group 1 (OR = 19.67) was much bigger than the OR of group 2 over group 1 (OR = 4.125), also suggesting that three risk groups (two cutoffs) was more appropriate than two (one cutoff). Subsequent calculations, on the training cohort that contained randomly selected 497 patients, with both the likelihood ratio test and the AUC test, put the two cutoff points at 0.32 and 0.97 (Table 5), with the former being close to 1/3, a signal for risk in clinical guidelines [11], and the latter close to 1, the fraction of complete penetration. Validation was performed with a testing cohort of 300 patients (Table 5). Thus patients with an invasion fraction less than 0.32 were at a low risk of LVSI, those between 0.32 and 0.97, medium risk, and those higher than 0.97, high risk. This finding may be of clinical importance.

**Table 5:** Multiple cutoff points: Depth of stromal invasion (in fraction) against risk of lymphovascular space invasion (LVSI).

Training (n = 497)			Testing (n = 300)				
Method	Cut 1	Cut 2	OR* 1	OR* 2	p 1	p 2	AUC
Log-rank test	0.32	0.97	5.085	39.38	0.037	<0.0001	0.73
Maximum AUC	0.32	0.97					

* OR: odds ratio. The reference for calculating the odds ratios was set at the low risk group.

### Comparison with traditional method, X^2^

To compare our package (Findcut) with traditional methods, we made use of the analysis of stromal invasion in response to LVSI. As analyzed above, the optimal cutoff points for stromal invasion was 2, at fractions of 0.32 and 0.97. We also used a traditional method, contingency table (X^2^), to perform similar analysis. However, unlike Findcut, traditional methods such as contingency tables (X^2^) usually require one to make a decision on how many cutoff numbers are needed before they can be employed. We thus conducted analyses with X^2^ when both one cutoff and two cutoffs were made. As shown in Table 6, when one cutoff was made, the testing results were quite unfavorable, while when two cutoffs were made, the results were comparable to Findcut. Therefore, although X^2^ can give similar results to our package, its performance is dependent on whether the number of cutoff points can be correctly guessed prior to analysis.

**Table 6:** Comparison of Findcut and X^2^: Stromal invasion vs. LVSI.

Methods	Training (n = 497)	Testing (n = 300)		
	Cutoff positions	sensitivity + specificity	AUC	Accuracy
X^2^, 1 cutoff	1	1.25	0.63	0.62
X^2^, 2 cutoffs	0.30, 0.88	1.33	0.68	0.66
Findcut, 2 cutoffs	0.32, 0.97	1.41	0.74	0.70

## Discussion

In clinical practice, stratification of patients is often useful for risk assessment or customized treatment plans. Although many algorithms and statistical packages are available to help researchers find one or more cutoff points, it is not always clear how many cutoffs are the best. In this study, we provided a package that enabled researchers to find the optimal number and location of cutoffs.

Log-rank test and likelihood ratio test were used to find optimal cutoffs in survival data, and they were both based on the most significant split, or the minimal p value. In case of dichotomized outcomes, likelihood ratio test based on the minimal p value was also used to find the best cutoffs. In addition, an AUC criterion that maximized the area under the ROC curve was also used. As described in the method section, maximizing AUC is similar to maximizing the classification quality of the model. Moreover, the AUC criterion is also similar to the Youden criterion which maximizes the sensitivity plus specificity. Furthermore, the package in this study can also serve as a framework to include other criteria incorporated by researchers.

We applied the package to the data obtained from cervical cancer. In 2012, there were 528,000 estimated cases of cervical cancer worldwide and 47,000 in the United States and European Union in spite of cytology screening, effective HPV screening, and introduction of vaccines in developed countries, and it was the leading cause of cancer-related deaths in developing countries [15]. The analyses of a few risk factors in relation to survival and prognosis in this study and their clinical relevance, therefore, may be of interest to gynecologists.

BMI is a good example of an optimal number of more than one cutoff points. Obesity has been associated with higher risk of death from cancer, including cervical cancer [12,13]. At the same time, several studies have also reported that extremely low BMI is also associated with poor prognosis and survival in cancer patients [14,16,17]. Therefore, it is desirable to find an optimal BMI interval. In this study, we used BMI as a covariate of survival, and found that in the events of both death and disease relapse, the optimal BMI for best survival was between 18.86 and 29.69, with the lower bound roughly corresponding to the commonly accepted limit for underweight (BMI = 18.5), and the higher bound, obesity (BMI = 30) [18]. Our result showed that optimizing patients’ weight may have positive effect on their survival.

In contrast to the example of BMI, classification of depth of stromal invasion into three risk groups stratified patients into low, medium, and high risk for having LVSI as patients with deeper stromal invasion were at a higher risk for LVSI. In clinical guidance for cervical cancer, an invasion fraction of 1/3 or higher is considered to present a risk for patients [11]. This threshold corresponds roughly with the upper boundary of the low risk group in our study (0.31, Table 5). We also found that the OR of high risk group over low risk group (OR2) was several times bigger than that of medium over low (OR1, Table 5), and the AIC values also favored two cutoffs. Both methods used here found the second cutoff point between medium and high risk to be at 0.96, a fraction at which the tumor penetrated the cervix stroma almost completely, presenting an alarming risk as the tumor may access other organs. This result may be helpful to gynecologists in evaluation of patients’ risk.

All the analysis in this study was performed when patients had been randomly grouped into a training cohort and a testing cohort, enabling us to validate our results with a separate set of data. In addition, we also made comparison to a traditional statistical method, X^2^, to illustrate the advantages of our package. A traditional method like X^2^ doesn’t provide any assistance to decide the optimal number of cutoff points and one must make a decision beforehand, and if that decision is incorrectly made, the cutoff points subsequently located will not be optimal. On the contrary, our package helps one with the decision first and the subsequent search for optimal locations will thus be well founded.

Many factors may contribute to the choice of an optimal number of cutoffs. Statistically, the AIC values can help researchers with the choice. The package developed here allows multiple cutoffs and facilitates optimal choice. We only considered cases with one and two cutoffs since these were the most common needs in clinical studies, however, the package can also handle more than 2 cutoffs when needs arise in practice. We recommend that clinical considerations should never be overlooked, and in most cases, should be the primary criteria when deciding how many cutoff points are optimal.

## References

[1] Mazumdar M, Glassman JR (2000) Categorizing a prognostic variable: review of methods, code for easy implementation and applications to decision-making about cancer treatments. Stat Med 19: 113–132. 10623917 10.1002/(sici)1097-0258(20000115)19:1<113::aid-sim245>3.0.co;2-o

[2] Perkins NJ, Schisterman EF (2006) The inconsistency of "optimal" cutpoints obtained using two criteria based on the receiver operating characteristic curve. Am J Epidemiol 163: 670–675. 10.1093/aje/kwj063 16410346 PMC1444894

[3] Budczies J, Klauschen F, Sinn BV, Gyorffy B, Schmitt WD, Darb-Esfahani S, et al (2012) Cutoff Finder: a comprehensive and straightforward Web application enabling rapid biomarker cutoff optimization. PLoS One 7: e51862 10.1371/journal.pone.0051862 23251644 PMC3522617

[4] Camp RL, Dolled-Filhart M, Rimm DL (2004) X-tile: a new bio-informatics tool for biomarker assessment and outcome-based cut-point optimization. Clin Cancer Res 10: 7252–7259. 10.1158/1078-0432.CCR-04-0713 15534099

[5] Lopez-Raton M, Cadarso-Suarez C, Rodriguez-Alvarez MX, Gude-Sampedro F (2014) OptimalCutpoints: An R Package for Selecting Optimal Cutpoints in Diagnostic Tests. Journal of Statistical Software 61: 1–36.

[6] T. R (2010) R: A language and environment of statistical computing. R Foundation for Statistical Computing: Vienna, Austria.

[7] Araújo A, Meira-Machado, Luís. (2015) Smooth Hazard Ratio Curves Taking a Reference Value. https://cran.r-project.org/web/packages/smoothHR/smoothHR.pdf.

[8] Therneau TM, Lumley, Thomas. (2016) Survival Analysis. https://cran.r-project.org/web/packages/survival/survival.pdf.

[9] Arbyn M, Castellsague X, de Sanjose S, Bruni L, Saraiya M, Bray F, et al (2011) Worldwide burden of cervical cancer in 2008. Ann Oncol 22: 2675–2686. 10.1093/annonc/mdr015 21471563

[10] Friedlander M, Grogan M (2002) Guidelines for the treatment of recurrent and metastatic cervical cancer. Oncologist 7: 342–347. 12185296

[11] Colombo N, Carinelli S, Colombo A, Marini C, Rollo D, Sessa C (2012) Cervical cancer: ESMO Clinical Practice Guidelines for diagnosis, treatment and follow-up. Ann Oncol 23 Suppl 7: vii27–32. 22997451 10.1093/annonc/mds268

[12] Xu X, Zhou L, Miao R, Chen W, Zhou Y, Pang Q, et al (2016) Association of cancer mortality with postdiagnosis overweight and obesity using body mass index. Oncotarget 7: 5023–5029. 10.18632/oncotarget.6517 26657291 PMC4826262

[13] Calle EE, Rodriguez C, Walker-Thurmond K, Thun MJ (2003) Overweight, obesity, and mortality from cancer in a prospectively studied cohort of U.S. adults. N Engl J Med 348: 1625–1638. 10.1056/NEJMoa021423 12711737

[14] Clark LH, Jackson AL, Soo AE, Orrey DC, Gehrig PA, Kim KH (2016) Extremes in body mass index affect overall survival in women with cervical cancer. Gynecol Oncol 141: 497–500. 10.1016/j.ygyno.2016.03.035 27058838

[15] Center MM, Jemal A, Lortet-Tieulent J, Ward E, Ferlay J, Brawley O, et al (2012) International variation in prostate cancer incidence and mortality rates. Eur Urol 61: 1079–1092. 10.1016/j.eururo.2012.02.054 22424666

[16] Matsunaga T, Suzuki K, Imashimizu K, Banno T, Takamochi K, Oh S (2015) Body Mass Index as a Prognostic Factor in Resected Lung Cancer: Obesity or Underweight, Which Is the Risk Factor? Thorac Cardiovasc Surg 63: 551–557. 10.1055/s-0035-1554964 26277079

[17] Kizer NT, Thaker PH, Gao F, Zighelboim I, Powell MA, Rader JS, et al (2011) The effects of body mass index on complications and survival outcomes in patients with cervical carcinoma undergoing curative chemoradiation therapy. Cancer 117: 948–956. 10.1002/cncr.25544 20945318 PMC4080792

[18] WHO (2006) BMI Classification. Global Database on Body Mass Index

